# Effects of *Helicobacter pylori* and Heat Shock Protein 70 on the Proliferation of Human Gastric Epithelial Cells

**DOI:** 10.1155/2014/794342

**Published:** 2014-08-05

**Authors:** Liping Tao, Hai Zou, Zhimin Huang

**Affiliations:** ^1^Department of Gastroenterology of the first Affiliated Hospital of Wenzhou Medical University, Wenzhou, Zhejiang 325000, China; ^2^Department of Hepatology, The Sixth Affiliated Hospital of Wenzhou Medical University, Lishui, Zhejiang 323000, China

## Abstract

Infection of *Helicobacter pylori (H. pylori)* changed the proliferation of gastric epithelial cells and decreased the expression of heat shock protein 70 (HSP70). However, the effects of *H. pylori* on the proliferation of gastric epithelial cells and the roles of HSP70 during the progress need further investigation. *Objective.* To investigate the effects of *Helicobacter pylori (H. pylori)* and heat shock protein 70 (HSP70) on the proliferation of human gastric epithelial cells. *Methods. H. pylori* and a human gastric epithelial cell line (AGS) were cocultured. The proliferation of AGS cells was quantitated by an MTT assay, and the expression of HSP70 in AGS cells was detected by Western blotting. HSP70 expression in AGS cells was silenced by small interfering RNA (*si*RNA) to investigate the role of HSP70. The *si*RNA-treated AGS cells were cocultured with *H. pylori* and cell proliferation was measured by an MTT assay. *Results.* The proliferation of AGS cells was accelerated by coculturing with *H. pylori* for 4 and 8 h, but was suppressed at 24 and 48 h. HSP70 expression was decreased in AGS cells infected by *H. pylori* for 48 h. The proliferation in HSP70-silenced AGS cells was inhibited after coculturing with *H. pylori* for 24 and 48 h compared with the control group. *Conclusions.* Coculture of *H. pylori* altered the proliferation of gastric epithelial cells and decreased HSP70 expression. HSP70 knockdown supplemented the inhibitory effect of *H. pylori* on proliferation of epithelial cells. These results indicate that the effects of *H. pylori* on the proliferation of gastric epithelial cells at least partially depend on the decreased expression of HSP70 induced by the bacterium.

## 1. Introduction

Gastric colonization by* H. pylori* occurs in more than half of all humans [1, 2], especially in Asia.* H. pylori* is an important pathogen which is associated with peptic ulcers and chronic atrophic gastritis, as well as gastric mucosa-associated lymphoid tissue lymphoma. It has also been defined as a class I carcinogen by the International Agency for Research on Cancer (IARC) [3–6]. However, the pathogenic mechanism by which* H. pylori* is involved in these diseases remains unclear.

As the front line of defense against the noxious action of ingested food or contaminating pathogens such as* H. pylori,* gastric mucosal epithelial cells must maintain stable cytokinetics and the balance between proliferation and apoptosis. Otherwise, development of atrophic gastritis, intestinal metaplasia, and even gastric carcinoma can occur. Infection by* H. pylori* has been reported to be associated with either increased or reduced rates of gastric epithelial cell proliferation and apoptosis both* in vivo* and* in vitro*, depending on the study [7–14].

The heat-shock system is one of the most important systems for maintaining the viability of the cell and its resistance to the damaging effects of various physiological and environmental stressors [15]. As a member of the heat-shock family, HSP70 is highly conserved, located in all cellular compartments including mitochondria, the endoplasmic reticulum, the cytosol, and the nucleus [16]. It is considered to play an important role in promoting proliferation and antiapoptosis [17–21]. Recent studies have indicated that specific downregulation of HSP70 in cells occurs in the presence of* H. pylori* [12, 22, 23]. However, there have been few studies on the role of HSP70 in the development of diseases of gastric epithelial cells infected by* H. pylori*. These findings raise the possibility that HSP70 might be involved in pathogenesis of diseases caused by* H. pylori,* but the role of HSP70 during the infection process and the relationship between* H. pylori*, HSP70, and gastric epithelial cells need further investigation.

In the light of these findings, we presume that the effects of* H. pylori* on gastric epithelial cells depend on the alteration of HSP70 expression induced by the bacterium. Therefore, we performed several experiments to elucidate the effects of* H. pylori* on gastric epithelial cells and its relationship with HSP70.

## 2. Materials and Methods

### 2.1. *H. pylori* Culture

The* H. pylori* strain (ATCC 700392, VacA^+^, CagA^+^) was provided by Professor Ning Dai, Sir Run Run Shaw Hospital, Zhejiang University, and grown on Columbia solid agar medium (OXOID, England), supplemented with 5% of fresh sheep's blood [24], and incubated in a microoxygen (5% O_2_, 10% CO_2_, and 85% N_2_) environment at 37°C for 48 h. The strain was confirmed during the culture. Before experimentation, bacteria were harvested and suspended in sterile PBS. The bacteria were counted using a spectrophotometer before administration to the cell culture, and suitable dilutions were prepared.

### 2.2. Cell Culture

The AGS human gastric epithelial cell line (CRL-1739, ATCC, USA) was provided by Zhejiang Key Laboratory of Traditional Chinese Medicine (TCM). Cells were grown in RPMI 1640 medium (Gibco, USA) which was supplemented with 10% fetal bovine serum, 100 *μ*g/mL streptomycin, and 100 U penicillin and incubated with 5% CO_2_ at 37°C. Before the experiments, 5 × 10^3^ of the AGS cells were seeded on 96-well plates and incubated overnight in RPMI 1640 medium, to which no antibiotics had been added. Cells were washed with sterile PBS before inoculation with the proper ratio of* H. pylori* or with RPMI 1640 alone. The cultures were incubated in a microoxygen (5% O_2_, 10% CO_2_, and 85% N_2_) environment at 37°C.

### 2.3. Effects of* H. pylori* on Proliferation of AGS Cells

AGS cells were cocultured with* H. pylori* at bacteria/cell ratios of 100 : 1, 200 : 1, and 500 : 1 for 4, 8, 24, and 48 h. Control cells were incubated in medium alone. At the end of each incubation, the cells were collected by trypsinization, and cell proliferation was measured by an MTT assay: MTT (5 mg/mL, Sigma) was added to each well, and the incubation was continued for 4 h at 37°C. Finally, the culture medium was removed and dimethyl sulfoxide (DMSO) was added to each well. The absorbance was determined with an ELISA reader at 490 nm [25].

### 2.4. Effects of* H. pylori* on Expression of HSP70 in AGS Cells

Cells were incubated with* H. pylori* (bacteria/cell ratio 200 : 1) or medium in absence of the bacteria for 48 h. After washing twice with PBS, the cells were harvested. Whole-cell extracts were prepared as described elsewhere. For Western blots, 100 *μ*g of protein samples was boiled with Western blot sample buffer and loaded on SDS-PAGE gels. After electrophoresis and transfer of the samples, the PVDF membrane (Millipore, Bedford, MA) was incubated with blocking buffer (5% nonfat dried milk in PBS) for 30 min at room temperature. This was followed by overnight exposure to primary antibody for HSP70 (diluted 1 : 4000) or *β*-actin (diluted 1 : 1000) (Santa Cruz, USA) and a 1-hour exposure to secondary antibody (diluted 1 : 5000) in blocking buffer. After each antibody probing, the membrane was washed three times for 10 minutes in TBST buffer. An ECL chemiluminescence reagent (Kibbutz Beit Haemek, Israel) was used to show the positive bands on the membrane [11].

### 2.5. Effects of* H. pylori* on Proliferation of HSP70-Silenced AGS Line

To clarify the effects of HSP70, we studied an HSP70-silenced AGS cell line which had been constructed previously in our laboratory. AGS cells which had been stably transfected with siRNAs of the control (siControl) or HSP70 (siHSP70) had a knockdown of HSP70 expression confirmed by RT-PCR and Western blot analysis (data not shown). In this study, siHSP70-transfected AGS cells and siControl-transfected AGS cells were cocultured with* H. pylori* (bacteria : epithelial cell ratio 200 : 1) for 4, 8, 24, 48, and 72 h. The siControl-transfected AGS cells were used as a control group. Cell proliferation was measured by an MTT assay as described above.

### 2.6. Statistical Analysis

**Figure 1:**
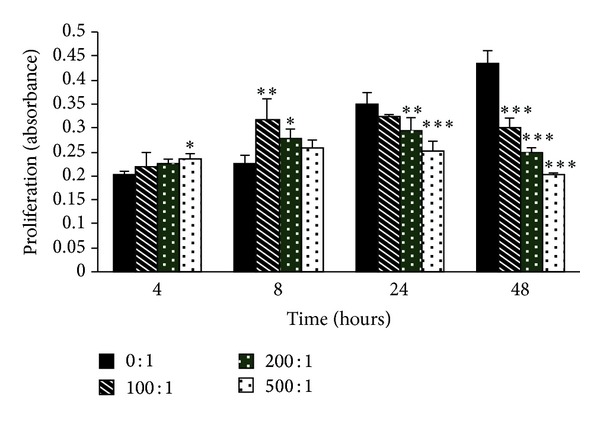
The effects of* H. pylori* on the proliferation of gastric epithelial cells. AGS cells were grown alone or with* H. pylori* (bacterium/cell ratio from 100 : 1 to 500 : 1) for 4, 8, 24, and 48 h. The proliferation of AGS cells was measured by an MTT assay. The results are expressed as absorbance at 490 nm. The data are shown as means ± SD (*P < 0.05, **P < 0.01, and ***P < 0.001 when compared with different groups, resp.).

Data are shown as means ± SD. The significance of the differences between the control group and the experimental group was evaluated by rank sum tests (Figure 1) and Student's *t*-tests (Figure 3).

## 3. Results

### 3.1. *H. pylori* Altered Proliferation of AGS Cells

The MTT assay showed that* H. pylori* altered cell proliferation in the AGS line, and the effect was dependent on the concentration of* H. pylori* as well as the duration of infection. After incubation for 4 and 8 h, cell proliferation in all of the study groups which were cocultured with* H. pylori* was significantly higher than that in control group, especially in the groups with a bacterium/cell ratio of 500 : 1 at 4 h (*P* < 0.05), 200 : 1 at 8 h (*P* < 0.05), and 100 : 1 at 8 h (*P* < 0.01). This indicated that* H. pylori* effects on cell proliferation in the AGS line occurred early after infection. However, after exposure for 24 h, cell proliferation in the study groups was inhibited, especially in the groups with a bacterium/cell ratio of 200 : 1 (*P* < 0.01) and 500 : 1 (*P* < 0.001), and more obviously in all of the study groups (*P* < 0.001). The inhibitory effects were concentration-dependent (Figure 1).

### 3.2. *H. pylori* Decreased HSP70 Expression in AGS Cells

**Figure 2:**
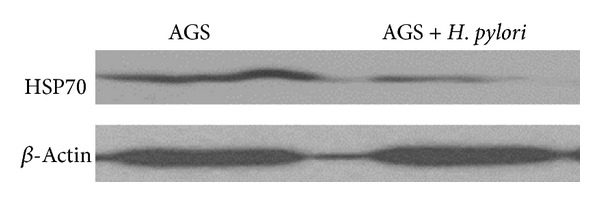
*H. pylori* effects on HSP70 expression in AGS cells. AGS cells were incubated with* H. pylori* (bacteria/cell ratio 200 : 1) or medium in absence of the bacterium for 48 h. The expression of HSP70 was measured by Western blotting. Anti-*β*-actin antibody was used to measure *β*-actin as a protein-loading control.

To investigate the molecular mechanism responsible for* H. pylori*-induced alteration of proliferation in AGS cells, HSP70 expression was measured in AGS cells infected by* H. pylori*. Addition of* H. pylori* to AGS cells significantly decreased HSP70 expression compared with cells which were grown in media with serum alone (Figure 2).

### 3.3. Proliferation of HSP70-Silenced AGS Line Exposed to* H. pylori*

**Figure 3:**
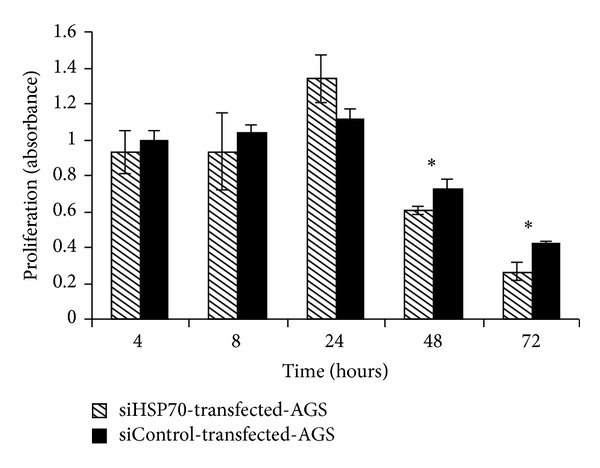
Proliferation of HSP70-silenced AGS line exposed to* H. pylori.* The HSP70-silenced AGS line (siHSP70-stably transfected-AGS cells) and control cells (siControl-stably transfected-AGS cells) were cocultured with* H. pylori* (bacteria : epithelial cell ratio 200 : 1) for the indicated times. Then, cell proliferation was detected by an MTT assay as described above. The results were expressed as absorbance. The data was shown as means ± SD (*P < 0.05, when compared with the control group).

The HSP70-silenced AGS line (siHSP70-stably transfected-AGS cells) and control cells (siControl-stably transfected-AGS cells) were incubated with* H. pylori* for 72 h. The MTT assay showed that, after coculturing for 48 and 72 h, proliferation in siHSP70-stale transfected-AGS cells was significantly lower than that in control group (*P* < 0.05) (Figure 3).

## 4. Discussion

Maintaining the balance between proliferation and apoptosis is important for gastric epithelial cells to maintain their normal function and prevent gastric diseases. Our data showed that* H. pylori* obviously changed the proliferation of gastric epithelial cells by transiently promoting proliferation at early time points and then inhibiting proliferation at later time points. This transition of the effects induced by* H. pylori* on the proliferation of gastric epithelial cells differs from the results of other researchers. Most of those authors showed that* H. pylori* inhibited proliferation in gastric epithelial cells [12, 13, 26–29], while others showed that* H. pylori* stimulated proliferation in gastric epithelial cells [8, 10, 11, 14, 30–32]. In addition, a few studies showed that the effect of* H. pylori* on cell proliferation was related to bacterial concentration. Xu et al. showed that low concentrations of* H. pylori* stimulated cell proliferation, while high concentrations inhibited proliferation and promoted apoptosis [25]. Ismail et al. found that low doses of outer membrane vesicles from* H. pylori* increased proliferation of AGS gastric epithelial cells, while, at higher doses, cell growth was arrested [33].

Our data showed that the inhibition on cell proliferation was not only related to infection time, but also related to the concentration of* H. pylori*. The variation of the results among this research may be due to the diversity of experiment design and methods, such as live* H. pylori*, culture filtrates, or* H. pylori* extracts.

In our study,* H. pylori* promoted proliferation of AGS cells at an early time which may be a stress reaction of cells to the inhibitor. Then, the effect was changed to suppress cell proliferation at a later time of* H. pylori* infection. This transition might be related to the time needed for the synthesis and secretion of virulence factors and related proteins of* H. pylori*.

HSP70 proteins act as molecular chaperones in the cell and play an important role in regulating cellular growth, promoting proliferation and antiapoptosis [34, 35]. In tumor cell lines, the increase in HSP70 expression has been shown to stimulate cell proliferation [36], while specific inhibition of HSP70 expression suppressed cell proliferation and increased apoptosis [37]. Our data showed that live* H. pylori* decreased HSP70 expression in gastric epithelial cells. This is consistent with a recent report which showed that HSP70 gene expression was specifically downregulated in gastric epithelium exposed to live* H. pylori* [12]. Konturek et al., using mice as their model for* in vivo* stomach infection with live* H. pylori*, had similar results [22]. These findings implied that HSP70 might participate in the pathogenic process caused by live* H. pylori*. In order to further understand the role of HSP70 in the development of diseases of gastric epithelial cells infected by* H. pylori*, we knocked down the expression of HSP70 in gastric epithelial cells by* siRNA*. Our data showed that HSP70 knockdown reinforced the inhibition effect of* H. pylori* on cell proliferation in gastric epithelial cells. This implies that the effect of* H. pylori* on the proliferation of gastric epithelial cells at least partially depends on the reduced expression of HSP70 induced by the bacterium. However, more studies are needed to investigate the exact relationship and the molecular mechanism.

In summary, our study indicates that* H. pylori* can change the proliferation of gastric epithelial cells, and this process is probably mediated by HSP70. The data may provide additional information for the molecular mechanism by which* H. pylori* causes diseases, such as chronic gastritis and even cancer.
